# Primary Ewing Sarcoma of the Cervical Spine: A Case Report and Literature Review

**DOI:** 10.7759/cureus.42687

**Published:** 2023-07-30

**Authors:** Omar M Shihadeh, Muhammad Mohsin Khan, Hayel Salih, Abdelnaser Thabet, Sirajeddin Belkhair

**Affiliations:** 1 Neurosurgery, Hamad Medical Corporation, Doha, QAT; 2 Neurosurgery, Weill Cornell Medicine-Qatar, Doha, QAT

**Keywords:** neurosurgery, spine oncology, ewing sarcoma, intradural extramedullary spine tumors, spine surgery

## Abstract

Ewing sarcoma is a rare neoplasm that mostly grows in bones or soft tissues around bones. Most cases of Ewing sarcoma occur in intra-skeletal locations (long bones, pelvis, or ribs) and rarely in extra-skeletal regions such as paravertebral or epidural space, whereas a primary intradural extramedullary Ewing sarcoma (IEES) is extremely rare. Fifty cases of primary IEES including our case were identified in the literature, so far, of which two-thirds are in the lumbosacral region, while only 12 reports described a cervical location of the tumor. Herein, we are presenting a case of C7-T1 primary IEES for a 24-year-old male, followed by a review of updated literature about the primary IEES in the cervical spine.

## Introduction

Ewing sarcoma is a malignant tumor, characterized by a poor prognosis and short survival rate, with the five-year survival rate being 60% [1]. Most cases occur in the long bones which are about 45% of the cases followed by the pelvis, or ribs but primary intradural extramedullary Ewing sarcoma (IEES) is extremely rare [2]. The most common differential diagnoses of intradural extramedullary spinal tumors are meningioma, schwannoma, neurofibroma, and metastasis. Unfortunately, all of these differentials share the same presentation and image findings with the primary IEES, which makes pre-operative radiological diagnosis difficult to achieve without histopathologic examination. The definite diagnosis requires immunohistochemistry analysis and/or cytogenetic studies to identify CD99 marker, (11;22)(q24;q12) translocation, or chimeric genes, like EWSR1-FLI1 [3].

Due to the rarity of this tumor, there is no consensus about its treatment guidelines. Surgery is currently the mainstay treatment in order to obtain a histological diagnosis and relieve compression of the cervical spinal cord due to the mass effect of the tumor. It is believed by some authors in the literature that patients who do not receive adjuvant treatment will have a poor prognosis in comparison with those who receive a combination of radiation and/or chemotherapy after surgery [4].

## Case presentation

A previously healthy 24-year-old male with a history of traumatic thoracic vertebral fracture four years prior to this presentation, came with progressive symptoms of upper back pain, right upper and lower limbs weakness to the extent of inability to walk and decreased sensations from nipples down over the last two weeks before presentation. In addition, he had difficulty passing urine for two days.

Physical examination revealed a significant motor weakness of the right upper and lower limbs; the power about three out of five, spastic lower limbs bilaterally, the sensory level at T4, increased patellar and Achilles tendon reflexes bilaterally, and the reflexes grade 3. Anal tone and perianal sensation were intact, and clonus and Hoffmann signs were not present.

**Figure 1:**
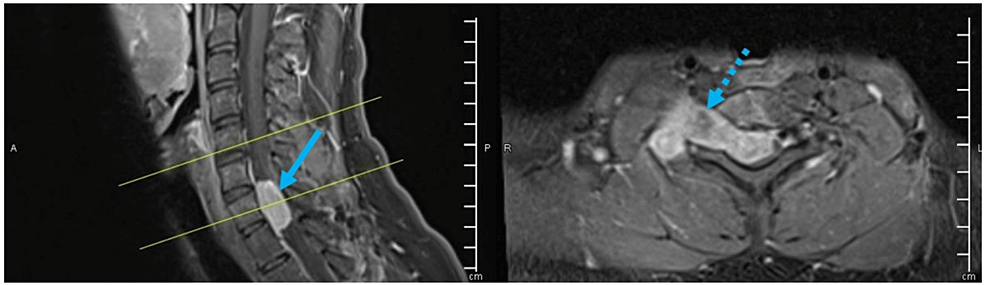
Pre-operative T1 weighted image with contrast, sagittal (left) and transverse (right). Well-defined intradural extramedullary homogenously enhancing lesion (solid arrow) at the level of C7-T1 compressing the spinal cord significantly toward the left side of the spinal canal and the lesion is extending toward and outside the right C7-T1 neural foramen with dumbbell-shaped appearance (dashed arrow). It measures approximately 20 x 48 x 42 mm in AP, transverse and craniocaudal dimensions and there is a widening of the right C7-T1 neural foramen.

**Figure 2:**
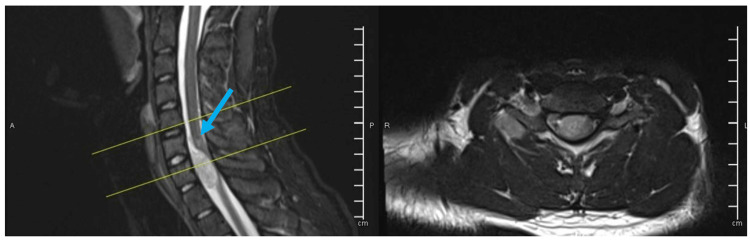
Pre-operative T2 weighted image, sagittal (left) and transverse (right). There is a mild intramedullary hyperintense T2 signal of the spinal cord at this level (blue arrow). The lesion follows the brachial plexus axis. The lesion also has a small projection/component surrounding the right C7 transverse process which shows an intramedullary hyperintense T2 signal and faint postcontrast enhancement; however, no cortical break was noted.

Magnetic resonance imaging (MRI) of the cervical spine with contrast showed a well-defined intradural, extramedullary homogenously enhanced lesion at the level of C7-T1 compressing the spinal cord significantly toward the left side of the spinal canal and the lesion is extending toward and outside the right C7-T1 neural foramen with dumbbell-shaped appearance, it measures approximately 20 x 48 x 42 mm in AP, transverse and craniocaudal dimensions and there is widening of right C7-T1 neural foramen (figure 1, 2).

An urgent C6, C7, T1 laminectomy with a right C7 facetectomy and resection of the lesion in the spinal canal then following the lesion extradurally through the right side C7-T1 foramen. The cervicothoracic spine was stabilized using C6 lateral mass screw and C7-T1 pedicle screws, neuromonitoring was used during the surgery. Intra-operatively the tumor was very vascular, greyish in color, and adherent to the surrounding spinal cord and nerve roots.

With significant tumor debulking and satisfactory decompression of the cervical cord, the were no intra-operative complications, post-operatively the patient showed dramatic improvement in the neurological symptoms, especially motor power.

**Figure 3:**
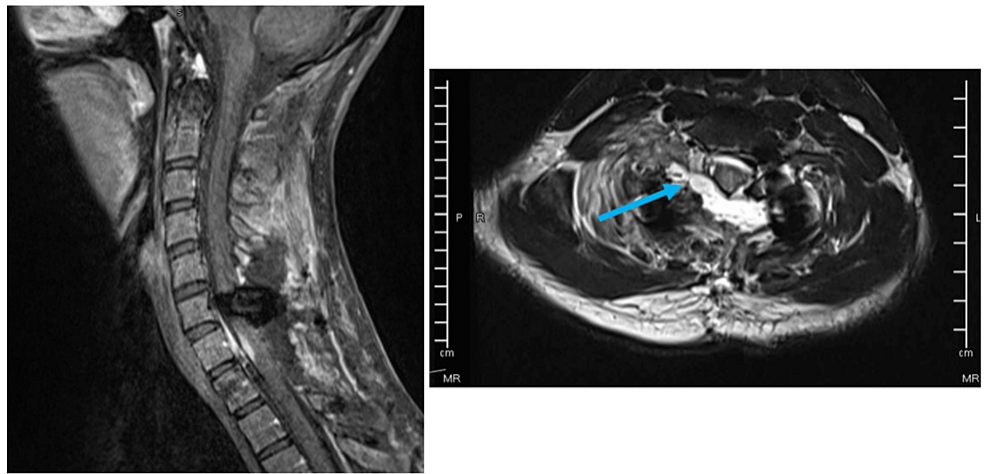
T1 weighted image with contrast, sagittal view (left) and post-operative T2 weighted image, transverse (right). Evidence of resection of intradural extramedullary mass lesion that was seen at the level of C7-T1 with resection of the right-sided extension into C7-T1 neural foramina with no definite enhancing residual at epidural component but there is suspected small residual extra spinally toward the chest (blue arrow).

MRI cervicothoracic spine with contrast was done one week after the surgery and showed evidence of complete resection of the intradural extramedullary mass lesion that was seen at the level of C7-T1 with resection of the right-sided extension into C7-T1 neural foramina with no definite enhancing residual at epidural component but there is suspected small residual extra spinally toward the chest (figure 3).

A whole spine and brain MRI did not show secondary lesions. Computed tomography (CT) of the chest, abdomen, and pelvis, and whole‑body positron emission tomography (PET) were negative for any tumors.

**Figure 4:**
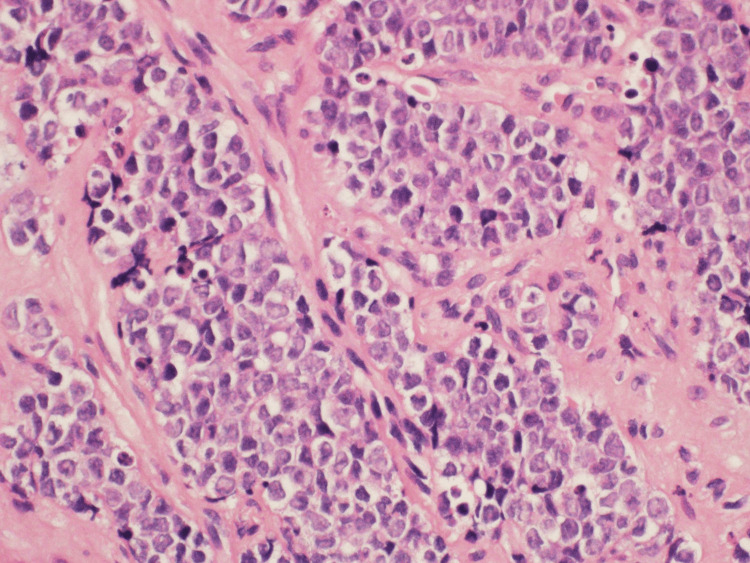
Microscopic view showing trabeculae and nests of malignant small round blue cell tumor (H&E×200).

**Figure 5:**
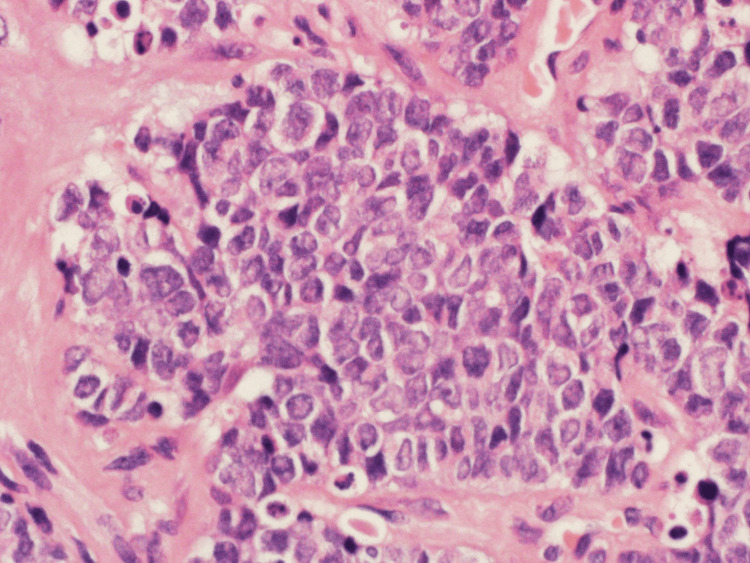
High power view showing details of the malignant small round blue cell tumor morphology (H&E×400).

Microscopic examination revealed a malignant small round blue cell tumor that was characterized by nests, trabeculae, and clusters (figure 4) of atypical small mesenchymal cells with ovoid nuclei, open chromatin, conspicuous nucleoli, few mitotic figures, and frequent apoptotic bodies (figure 5).

**Figure 6:**
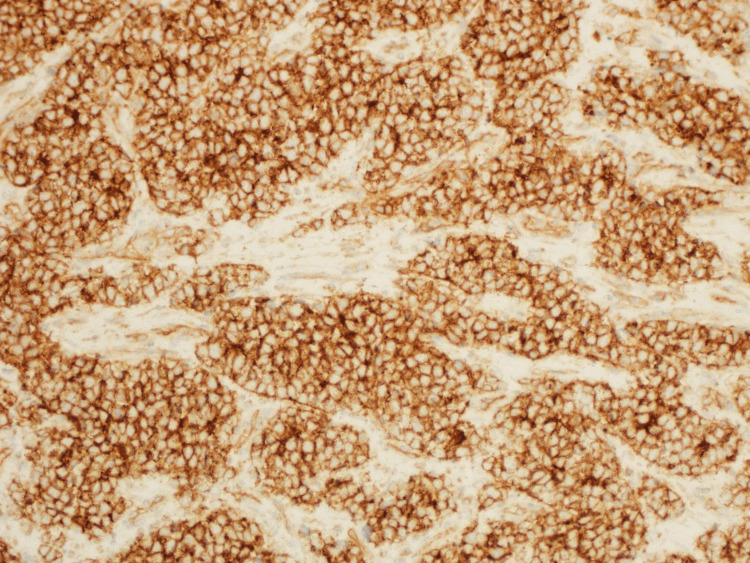
Immunohistochemistry view showing strong positivity of the tumor cells with CD99 (immunohistochemistry×200).

A panel of immunohistochemical antibodies was performed and showed the tumor cells to be positive with CD99 (figure 6), BCL-2, FLI-1, TLE-1, ERG, and INI-1; while negative with CD34, myogenin, MyoD1, chromogranin, Synaptophysin, S100, CD45, Sox-10, SAT-B2, CK AE1/AE3, TTF-1, CD56, desmin, EMA and CK 7. Ki-67 index is 20%. Fluorescence in situ hybridization (FISH) had been performed and showed EWSR1 rearrangement confirming the morphological diagnosis of Ewing’s sarcoma.

We discussed his condition in our multidisciplinary teams meeting regarding adjuvant treatment and decided to offer him adjuvant chemotherapy and local radiotherapy at the region of the tumor. Indeed, the patient was started on VAC/IE chemotherapy protocol (vincristine, Adriamycin, and cyclophosphamide followed by ifosfamide and etoposide). The patient in general tolerated chemotherapy with no complications apart from a short duration of significant drop of the absolute neutrophil count (ANC) discovered on routine labs, following which filgrastim was given. For a few months after the surgery, patient continued to improve clinically. Unfortunately, after 6 months of treatment, he decided not to complete his medical course of chemoradiotherapy and wanted to go back to his home country. One month later he presented with acute hydrocephalus, ventriculoperitoneal (VP) shunt was inserted, two weeks later (7 months since time of diagnosis) he passed away.

## Discussion

We had done a thorough literature review of the cases of Ewing sarcoma involving the cervical spine. A total of 11 confirmed cases of primary intradural extramedullary Ewing sarcoma/peripheral primitive neuroectodermal tumor in the cervical spine were identified in the literature. Our reported patient will be case number 12. The collective and individual data presentations of the literature review are detailed in Table 1 and Table 2 respectively.

**Table 1:** Collective data presentation of the literature review. STR: Sub-total resection, GTR: Gross total resection

Characteristics	Value
Cohort size (n)	12
Age (years)	
Median age	29.5
Range of ages	(10-39)
Gender (n, %)	
Male	5, 42%
Female	7, 58%
Chief symptoms (n, %)	
Paresthesia	11, 92%
Motor weakness	10, 83%
Neck/back pain	6, 50%
Urinary dysfunction	4, 33%
Duration of symptoms (weeks)	
Range of durations	(2-12)
Median duration	5
Diagnosis (n, %)	
Histopathology	12, 100%
Immunohistochemistry	10, 83%
Cytogenetics	7, 58%
Treatment (n)	
Surgery (GTR/STR)	12 (4/7)
Chemotherapy	8
Radiotherapy	7
Prognosis	
Progression-free survival range	10 days - 36 months
Recurrence rate (%)	63%
Overall survival range	(1-48) months
Death rate	33%

**Table 2:** Individual data presentation of the literature review. M: Male, F: Female C: Cervical, T: Thoracic IHC: Immunohistochemistry FISH: Fluorescence in situ hybridization RT-PCR: Reverse transcription polymerase chain reaction STR: Sub-total resection, GTR: Gross total resection VAIA: Vincristine, actinomycin-D (dactinomycin), ifosfamide, and Adriamycin (doxorubicin) IFO: Ifosfamide CTX: Cyclophosphamide, THP: Pirarubicin, VCR: Vincristine VDC/IE: Vincristine, doxorubicin, and cyclophosphamide/ifosfamide and etoposide VAC/IE: Vincristine, Adriamycin, and cyclophosphamide/ifosfamide and etoposide VACM: Vincristine, Adriamycin, cyclophosphamide, and mesna Duration of symptoms: Starting from the onset of the symptoms till the first presentation Progression-free survival: The length of time during and after the treatment of a disease, that a patient lives with the disease but it does not get worse

No	Author	Age, sex	Location	Presentation		Diagnosis			Treatment			Prognosis		
				Chief complaints	Duration of symptoms	Histopathology	IHC	Genetics (FISH/RT-PCR)	Surgery (intraoperative findings)	Chemotherapy (protocol)	Radiotherapy (dose)	Progression-free survival	Recurrence	Overall survival
1	Uesaka, 2003 [5]	11, F	C7-T1	Back pain, progressive paresthesias, weakness in both lower limbs	4 weeks	Yes	CD99	No	STR (adhered to the right posterior C8 nerve root, intratumoral hematoma)	N/A	N/A	N/A	N/A	N/A
2	Harimaya, 2003 [6]	30, F	C2-C4	Numbness of the extremities, urinary retention	6 weeks	Yes	No	No	STR (N/A)	Yes (VAIA)	Yes (50 Gy)	4 months	yes	14 months
3	Woestenborghs, 2005 [7]	11, M	C4-T2	Progressive quadriparesis	N/A	Yes	CD99, vimentin	t(11;22)(q24;qI2)	STR (highly vascular, invading the dorsal side of the spinal cord at C5-C6 and several nerve roots)	Yes (Euro-EWING 99 protocol)	N/A	N/A	N/A	N/A
4	Kim, 2009 [8]	32, F	C3-C5	Progressive paresis, pain, and numbness of the upper limbs	6 weeks	Yes	No	No	STR (adhered to the posterior nerve roots)	Yes (etoposide and ifosfamide)	Yes (30 Gy)	12 months	N/A	N/A
5	Yan, 2011 [4]	10, M	C2-C3	Neck pain, worsening muscle weakness, dysuria	3 weeks	Yes	CD99, vimentin	No	GTR (moderate blood supply, intact pseudo capsule)	No	No	N/A	Yes	1 month
6	Gong, 2015 [9]	39, F	C4-C6	Left upper limb pain, numbness	12 weeks	Yes	CD99	t(11;22)(q24;qI2)	GTR (soft consistency)	Yes (CTX+THP+VCR)	Yes (38 Gy)	36 months	Yes	N/A
7	Bostelmann, 2016 [10]	29, M	C7-T1	Right C7-radiculopathy progressed to hemiparesis	12 weeks	Yes	CD99, vimentin, cytokeratin, synaptophysin.	t(11;22)(q24;qI2)	GTR (N/A)	Yes (vincristine, ifosfamide, doxorubicin, etoposide)	Yes (36 Gy)	1 month	Yes	18 months
8	Chihak, 2016 [1]	25, M	C4-C7	Numbness, weakness of the right hand	4 weeks	Yes	CD99	t(11;22)(q24;qI2)	STR (N/A)	Yes (VDC/IE)	Yes (54 Gy)	20 months	No	20 months
9	Tan, 2019 [11]	34, F	C4-T3	Neck pain, numbness, quadriparesis, urinary retention	8 weeks	Yes	CD99, CD56	t(11;22)(q24;qI2)	STR (moderately vascular, firm consistency, engulfing the nerve roots and adherent to the spinal cord)	No	Yes (46 Gy)	10 days	Yes	11 months
10	Warade, 2019 [12]	33, F	C6-C7	Neck pain, paraesthesia in the right upper limb	4 weeks	Yes	CD99	t(11;22)(q24;qI2)	GTR (well encapsulated, soft consistency, extremely vascular, engulfing C7 nerve root)	Yes (VACM)	Yes (40 Gy)	6 months	No	N/A
11	Ganapathy, 2020 [13]	33, F	Clivus-C3	Features suggestive of high cervical compressive myelopathy, C3 radiculopathy	N/A	Yes	FLI‑1, CD99	No	N/A	No	No	N/A	N/A	48 months
12	Shihadeh, 2021	24, M	C7-T1	Right limbs weakness, paraesthesia, neck pain, urinary retention	2 weeks	Yes	CD99	t(11;22)(q24;qI2)	STR (highly vascular, adherent to the surrounding spinal cord and nerve roots)	VAC/IE	Not yet	4 months	Yes	7 months

Typically, young adults are affected (ages range from 10 to 39 years; median age is 30 years). Of these 12 cases, 7 were females (58%) and 5 were males (42%). Clinical presentation is commonly characterized by paraesthesia (11 cases), motor weakness (10 cases), neck/back pain (6 cases), and urinary dysfunction (4 cases). The duration of symptoms before diagnosis was over a short period of time, which ranges from 2 to 12 weeks and a median duration of 6 weeks. All 12 patients underwent urgent surgical decompression and resection of the lesion. Gross total resection was achieved in only 4 cases (36%) and this is largely because the tumor is usually adherent to the surrounding nerve roots/spinal cord or sometimes engulfs or even invades them in addition to the finding of moderate to high vascularity, which was reported in 5 cases; all of these findings are in accordance with our intra-operative findings as well as Tan et al. and Lu et al. in their systemic reviews [11, 14]. All reported cases have made the initial diagnosis of Ewing sarcoma via histopathology where the typical histological appearance comprises primitive dense sheets of small round cells with high nuclear/cytoplasmic ratio and uniform nuclei with fine chromatin in which Homer Wright rosettes were sometimes reported. This diagnosis was confirmed subsequently using immunohistochemistry in 10 cases, including ours, which always showed positivity of CD99 and sometimes vimentin markers. Cytogenetics was used for further assertion in 7 cases, including our case, through using reverse transcription polymerase chain reaction (RT-PCR) or FISH. They showed either t(11;22)(q24;qI2) or chimeric gene (EWSR1-FLI1 or EWSR1-ERG), which are specific for the diagnosis of Ewing sarcoma. In terms of treatment, this review emphasizes the importance of early surgical decompression of the cervical spinal cord which has been carried out in all cases as the first-line treatment. On the other hand, the review does not show complete agreement regarding the subsequent management plan of chemoradiotherapy. Eight cases have been treated with adjuvant chemotherapy and the most commonly used protocol was vincristine, doxorubicin, and cyclophosphamide in alteration with ifosfamide and etoposide (VDC/IE) which is reported by Takami et al. [15]. Neoadjuvant radiotherapy was initiated in 7 cases also with a radiation dose ranging from 30 to 54 Gy. Some cases reported emergency neoadjuvant radiotherapy due to early decompensation. Some cases have been exposed to craniospinal axis radiation, while others exposed to only localized radiation therapy. Although limited by a small sample size, Chihak et al. reported improved survival and absence of recurrence in patients who received craniospinal radiation over those who received local therapy [1].

This pooled review of reported cases showed a very poor prognosis for this tumor which is in accordance with the other reviews done previously. Progression-free survival was ranging from 10 days to 36 months. Overall survival ranges from 1 month to 48 months.

Ewing sarcoma is a high-grade small round cell tumor of neuroectodermal origin and it shares the presence of a common chromosomal translocation with other tumors like peripheral primitive neuroectodermal tumor (pPNET), atypical Ewing sarcoma, and Askin tumor [13]. Most cases of Ewing sarcoma occur in the skeleton (the long bones, pelvis, or ribs) and rarely in extra-skeletal regions such as the paravertebral or epidural space, whereas the primary IEES is extremely rare [2]. it shares similar clinical pictures and radiological images with the most common differential diagnoses for intradural spinal tumors like meningioma, nerve sheath tumors such as schwannoma or neurofibroma, and metastasis. The definitive diagnosis of primary IEES requires immunohistochemistry analysis and cytogenetic studies to identify the (11;22)(q24;q12) translocation [3]. In light of this, molecular confirmation by either FISH or RT-PCR is mandatory, but, unfortunately, there were several reports in which the diagnosis was based solely on histopathology and immunohistochemistry. The diagnosis could not be confirmed in such cases, although they were reported as Ewing’s sarcoma including 5 cases in our review which did not report confirmation by using cytogenetic studies. Pathogenesis is unclear but EWS/ETS fusions are thought to be the presumed initiating oncogenic event which is required for the proliferation and tumorigenesis through the promotion of abnormal cellular growth by transcriptionally modulating a network of target genes [16]. IEES appears to have a poor prognosis with only 57.6% crude two-year event-free survival in patients treated with multimodality therapy [1]. Lu et al.'s systemic review of 45 cases of IEES of all spinal levels reported an overall recurrence of 46%, a median progression-free survival of 12 months, and a median overall survival of 14 months [6]. Five-year overall survival was 40%, which is much worse than that of extraosseous soft tissue Ewing’s sarcomas (70%) or even the osseous lesions (62%) [17].

Surgery is currently the mainstay treatment for IEES because it is often difficult to differentiate Ewing sarcoma or pPNET from other diseases in the spinal canal, including schwannoma and meningioma. Therefore, a pathological examination after surgery is essential for making the initial diagnosis. Secondly, reducing the tumor volume by surgical resection is the basis for other adjuvant treatments, including radiotherapy and chemotherapy. According to the previously reported cases, patients who do not receive adjuvant treatment show a poor prognosis [4], whereas a combination of radiation and/or chemotherapy with surgery leads to better results [18]. Some authors favor entire craniospinal axis radiotherapy, similar to the standard treatment for central primitive neuroectodermal tumor (cPNET) instead of focal radiotherapy to the primary site which they believe associates with a high recurrence rate [1]. the current recommendations for osseous Ewing sarcoma, which is suggested by some authors as the initial regime for the primary IEES, include alternating cycles of vincristine, doxorubicin, and cyclophosphamide with ifosfamide and etoposide [19]. The rarity and limited evidence of the therapeutic aspects of IEES rendered the standard treatment guidelines for these tumors vague. In addition to that, radiotherapy is limited by the tolerance of the spinal cord (55Gy), and achieving tumor-free surgical margins or en-bloc resection may be difficult due to the surrounding anatomic structures.

## Conclusions

In summary, we added a rare case of cervical intradural extramedullary Ewing sarcoma with a comprehensive review of the previously reported cases of IEES in the cervical spine. This review highlights the importance of considering Ewing sarcoma as one of the differential diagnoses of intradural extramedullary lesions of the spinal cord. Early surgical decompression is necessary to improve neurological function, although gross total tumor resection is mostly difficult to achieve; it should be followed by adjuvant therapy. 
